# Understanding Podocyte Biology to Develop Novel Kidney Therapeutics

**DOI:** 10.3389/fendo.2018.00409

**Published:** 2018-07-23

**Authors:** Mark A. Lal, Jaakko Patrakka

**Affiliations:** ^1^Bioscience, Cardiovascular, Renal and Metabolism, Innovative Medicines Biotech Unit, AstraZeneca, Gothenburg, Sweden; ^2^Karolinska Institutet/AstraZeneca Integrated Cardio Metabolic Center, Department of Laboratory Medicine, Karolinska Institutet at Karolinska University Hospital Huddinge, Stockholm, Sweden

**Keywords:** podocyte, diabetic nephropathy, glomerulus, targeted therapy, chronic kidney disease

## Abstract

Over the past two decades it has become increasing clear that injury and loss of podocytes is an early and common clinical observation presented in many forms of glomerulopathy and chronic kidney disease. Identification of disease-causing monogenic mutations in numerous podocyte-expressed genes as well as studies conducted using preclinical animal models have shown that the podocyte plays a central role in establishing kidney dysfunction. In this review, we summarize current knowledge regarding the potential for podocyte-targeted therapies and give our view on how a deeper understanding of the molecular makeup of the podocyte will enable future therapeutic interventions. Specifically, we recount some of the currently described podocentric strategies for therapy and summarize the status and evolution of various model systems used to facilitate our understanding of the molecular and functional underpinnings of podocyte biology.

## Introduction

Glomerular capillary tufts are responsible for renal ultrafiltration of blood and formation of primary urine. The filtration barrier is formed of three layers: highly fenestrated glomerular endothelial cells, the glomerular basement membrane (GBM) and podocyte cells with their foot processes. Mesangial cells, that are considered to act as pericytes in the glomerulus, are the third cellular component of glomerular tufts.

**Figure 1:**
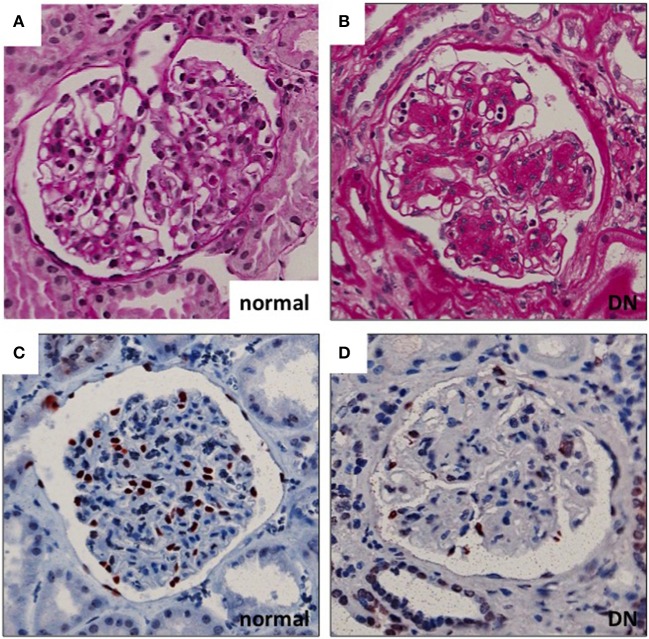
Glomerular histopathology in DN. **(A,B)** Diabetic glomeruli present nodular expansion of mesangial matrix as detected by periodic acid-Schiff staining. **(C,D)** The number of podocytes is decreased in DN glomeruli as demonstrated by wt1 staining (marker for podocyte nuclei). Magnifications: ×200.

Diabetic nephropathy (DN), one of the microvascular complications of diabetes, affects 20–40% of patients with diabetes mellitus (1). Renal glomeruli are the main targets of injury in the disease. Glomerular hyperfiltration, mediated at least partially by tubulo-glomerular feedback, is considered an early pathogenic event in the development of DN. This is followed by albumin leakage through the glomerular filtration barrier (GFB) (resulting in albuminuria), which is the hallmark clinical sign of DN. Histologically, glomeruli show thickening of the GBM, mesangial cell activation with expansion of mesangial matrix and podocyte damage with eventual loss of podocytes (Figure 1). Podocyte density is one of the best predictors of the progression in DN. This is related to the fact that podocytes are terminally differentiated and cannot replicate or regenerate significantly (at least) in adults (2). The number of podocytes seems to be very critical for kidney health as in animal models the loss of >20% of podocytes results in irreversible glomerular damage that progresses to end stage renal disease (3). Thus, the extent of podocyte damage and loss seems to define the progression rate in many kidney diseases.

Numerous studies in transgenic mouse models developed to express/inactivate single genes exclusively in podocyte cells, with the use of podocyte-specific promoters, have validated the podocyte as an attractive therapeutic target of therapy in DN. For instance, kidneys, in which epidermal growth factor receptor (EGFR) has specifically been deleted in podocytes, are protected from diabetic injury and the progression of DN is interrupted (4). These studies demonstrate that manipulation of podocyte signaling in a cell-specific manner is sufficient to stop the progression of nephropathy and unveils an obvious platform for exploring future treatment options.

While the progression of acquired glomerular diseases, such as diabetic kidney disease, is a result of the convergence of multiple injurious pathways, a deep and thorough understanding of the pathomechanisms initiating and driving podocyte-specific hereditary diseases may hold promise to identify and discriminate among those potential targets that might be best suited to interrupt the development of more complex acquired glomerulopathies. Over 50 monogenic glomerulopathies have been described and understanding the molecular mechanisms of their causative roles in mediating podocyte dysfunction in glomerular disease will hopefully provide us with the knowledge to realize the potential for manipulating pleiotropic gene function in non-hereditary glomerular disease. Most of these monogenic targets themselves are not pharmacologically tractable candidates, yet understanding the responsible mechanisms accounting for their roles in disease etiology may very well identify key secondary mediators that can be manipulated. In the following section, we provide examples of various therapeutic strategies where the podocyte may be considered the principal target cell involved in the differing modes of action.

## Podocentric therapeutic options for DN and other glomerulopathies

### ACE inhibitors and ARBs

Today, patients with DN are treated with either angiotensin-converting enzyme (ACE) inhibitors or angiotensin receptor blockers (ARB). Both drugs reduce the glomerular filtration pressure by primarily inhibiting efferent arteriole vasoconstriction. Based on animal and cell culture studies, there is considerable evidence suggesting that these drugs may also partially mediate their beneficial effects via direct actions on cells of the glomerulus, including podocytes (5, 6). However, data questioning a definitive physiological role for the main angiotensin receptor, angiotensin II receptor type 1 (AT1R), in podocytes also exists. In fact, in mice with podocyte-specific AT1R deletion, angiotensin inhibition with both ACE and ARB continues to afford podocyte protection and prevents the development of glomerulosclerosis (7). This finding suggests that the principal cellular mechanisms accounting for the established efficacy of AT1R inhibition under this setting are unlikely to directly involve podocytes expressing AT1R. Additional studies are needed to expand upon these findings in order to better define the individual cellular targets of today's standard of care treatment. Ultimately, it will be important to evaluate and utilize the gene expression pattern of individual cells of the glomerulus and kidney using technologies such as single cell RNA sequencing so we can better couple molecular and therapeutic activities to the correct target cell (8, 9).

### Glucocorticoids

Glucocorticoids are the mainstay of treatment for minimal change disease, the most common cause of nephrotic syndrome in children. These patients typically respond robustly to this treatment as massive albuminuria disappears in a few days after onset of glucocorticoid treatment. This outcome has traditionally been considered to be due to immunosuppressive effects, although recently, two interesting studies suggest otherwise (10, 11). Kuppe et al. inactivated the glucocorticoid receptor in kidney epithelial cells (including podocytes) and found that mice were partially protected from podocyte loss in a model of glomerulonephritis and that this effect was very similar to that seen following systemic glucocorticoid therapy. Moreover, the addition of this therapy to conditional knockout animals did not add any beneficial effects. Thus, at least in this animal model, kidney epithelial cells (including podocytes), seemed to mediate the kidney-protective effects of glucocorticoids. The study by Mallipattu et al. investigated the involvement of Kruppel-like factor 15 (KLF15), a kidney-enriched transcription factor having a role in podocyte differentiation and response to injury, in mediating the response of podocytes to glucocorticoids. In this study, the authors showed that podocyte-specific inactivation of KLF15 markedly reduced the anti-proteinuric effects of glucocorticoids in LPS, adriamycin and anti-glomerular antibody induced nephropathy models. Taken together, glucocorticoids appear to have direct, non-immune mediated, reno-protective effects and it is likely that podocytes are one of the main targets of this action.

### Calcineurin inhibitors

Calcineurin inhibitors have been shown to have direct effects on podocytes (12). This study showed that the calcineurin-dependent dephosphorylation of the podocyte foot process protein, synaptopodin, results in an instable podocyte actin cytoskeleton. Treatment of mice with ciclosporin thus promoted phosphorylation of synaptopodin, stabilization of actin cytoskeleton and diminished proteinuria. Importantly, these effects were independent of the immune system.

### Rituximab

Rituximab, a monoclonal antibody directed against CD20 expressed on the surface of B-cells, has been used successfully to treat various glomerulopathies in which podocytes are injured (13). Due to its B-cell depleting properties, the drug is used in a wide variety of immune-mediated diseases including rheumatoid arthritis. Fornoni et al. suggested that rituximab may have direct effects on podocytes by binding to sphingomyelinase-like phosphodiesterase receptor (SMPDL-3b) (14). Through this receptor, rituximab was proposed to maintain intact actin cytoskeleton structure and in that way have kidney-protective effects. A recent study, however, by Shaw and colleagues highlights the importance of B-cells *per se* in mediating the effects of rituximab in glomerular disease (15). In this study, B-cell derived IL-4 results in podocyte damage and proteinuria in mouse and activation of IL-4 signaling in glomeruli is observed in a significant proportion of patients with glomerular disease. This supports the idea that the anti-proteinuric effects of rituximab in the glomerulus are mediated mainly by B-cells. Although this study gives interesting new insight into the pathogenesis of glomerular diseases, more studies need to be done in other disease models to understand the contribution of B-cell derived cytokines in the glomerulus.

### B7-1 (CD80)

B7-1 (CD80) is a transmembrane protein mostly known as a costimulatory receptor on antigen presenting cells. Reiser et al. reported in 2004 that B7-1 expression was induced in podocytes in a mouse model of proteinuria and that this had a pathogenic role as B7-1 deficient animals were protected from proteinuria (16). In 2013, a follow-up study detected B7-1 expression in podocytes in a number of human proteinuric diseases and excitingly, a B7-1 blocker abatacept was used to successfully treat a small cohort of patients with a resistant form of recurrent focal segmental glomerulosclerosis (FSGS) (17). Therefore, it was proposed that B7-1 expression in podocytes could be a biomarker to identify proteinuric patients who could benefit from treatment with abatacept. Moreover, elevated B7-1 expression in podocytes was demonstrated in about 50% of kidney biopsy specimens from patients with DN (18). The expression levels seemed to correlate with the severity of glomerulopathy and treatment of diabetic mouse models with CTLA4-Ig, an inhibitor of B7-1 pathway, protected podocytes and diminished proteinuria.

These initial encouraging results have been followed by many studies that have questioned the value of B7-1 expression in podocytes as a biomarker and as a therapeutic target. In patients with minimal change disease, FSGS and DN, no B7-1 expression was detected in podocytes (19, 20). In mouse models of proteinuria, no B7-1 expression was detected in podocytes (21). Similarly, treatment of patients with recurrent FSGS in transplants with abatacept failed to give any beneficial effects (22). The reasons for these discrepancies is unclear. It is possible that the protective effect of B7-1 inhibition in the original mouse studies was mediated by B7-1 expression in immune cells. In fact, B7-1 expression is detected in infiltrating immune cells in the glomerulus in many proteinuric mouse models. One other possible explanation for the different responses to abatacept treatment in FSGS is that the disease is very heterogenous; it may be that only a small subset of FSGS patients responds to abatacept. Unfortunately, to identify that group of responders seems to be difficult, as the detection of B7-1 expression using immunohistochemistry is challenging and currently a matter of controversy.

### TRPC-channels

Gain-of-function mutations in the transient receptor potential cation channel TRPC6 are associated with the onset of familial forms of FSGS and increased expression of this channel has been described in acquired glomerular disorders including DN (23, 24). TRPC6 localizes to podocyte major and foot processes as well close to the slit diaphragm where it interacts with nephrin and podocin (but not CD2AP) (24). Just how TRPC6 channel activity determines podocyte structure and function is the subject of intense investigation. As a receptor channel, unraveling the TRPC6-dependent effects in the initiation of podocyte injury are particularly interesting. Activating mutations of TRPC6 lead to calcium influx and podocyte stress leading to the initial idea that pharmacological inhibition of TRPC6 may protect podocytes and prevent disease progression. Notably, TRPC6 knockout mice do not present a glomerular phenotype whereas mice overexpressing podocyte-specific mutant or wt TRPC6 develop albuminuria (25). The story is however more complicated as the role of TRPC6 in determining podocyte health appears context-dependent and temporally biphasic depending on the underlying pathophysiology (26). As such it is not clear whether pharmacological inhibition of TRPC6 represents a general mechanism to promote podocyte health in DN. Although lacking a genetic link to human glomerular disease, a second TRPC channel, TRPC5 may also be an important player in determining podocyte function in health and disease. Global TRPC5 knockout mice are protected against acute podocyte injury and TRPC5 small molecule inhibitors have been recently shown to suppress albuminuria in both a transgenic model of podocyte-driven glomerular injury as well as Dahl salt-sensitive rats (27, 28). These results highlight the therapeutic potential for targeting this channel but questions remain as to whether the podocyte is in fact the primary target. Human target validation for TRPC5 is limited and overexpressing either wild-type TRPC5 or a TRPC5 ion-pore mutant in mice did not result in podocyte injury or proteinuria well into adulthood or after podocyte injury (29). Further studies are needed to clarify the integrated roles of the TRPC family of channels in podocyte biology.

### CoQ10

Mutations in enzymes involved in coenzyme Q10 biosynthesis, such as Coq2, Coq6 and aarF domain containing kinase 5 (ADCK4), have been identified by whole exome sequencing in few families with hereditary glomerular disorders (30–32). The clinical picture of these disorders varies from multi-organ involvement to isolated nephrotic syndrome. Coq2, Coq6 and ADCK4 are highly expressed by podocytes suggesting that deficient CoQ10-function in podocytes is one of the drivers of disease development. This is supported by findings in cell culture, zebrafish and drosophila studies. Importantly, patients with defective CoQ10 biosynthesis responded to CoQ10 supplementation, suggesting that this therapy is effectively targeting and protecting podocytes (30). CoQ10 is essential for the mitochondrial respiratory chain and controls production of reactive oxygen species. It will be interesting to see how these pathways are involved in the pathogenesis of common glomerular diseases.

### Dynamin

Dysregulation of the actin cytoskeleton in podocyte foot processes is a central mediator in the pathogenesis of proteinuria. This is best highlighted by studies in hereditary proteinuric diseases as disease-causing mutations have been identified in several molecules involved either directly or indirectly in the regulation of the podocyte actin cytoskeleton (33, 34). Targeting the actin cytoskeleton in podocytes could thus be one possibility to develop new therapeutic options for DN and as evidenced by recent studies of causative mutations in nephrotic syndrome, an ever expanding repertoire of actin-modulating targets can be considered for therapeutic manipulation (35). Below, we highlight the example of dynamin regulation as an approach to modulate podocyte actin cytoskeleton dynamics.

Dynamin is a GTPase regulating endocytosis and actin cytoskeleton assembly. Dynamin oligomerizes in an actin-dependent manner and promotes actin polymerization via direct interaction. It has been shown to be essential for podocyte structure and function. A small molecule, Bis-T-23, has previously been shown to augment dynamin oligomerization in cultured podocytes. Schiffer et al. treated various rodent models of proteinuria with Bis-T-23 and interestingly showed that the treatment ameliorated proteinuria and development of histopathological changes, supporting the idea that the podocyte actin cytoskeleton can be directly targeted to treat proteinuric diseases (36). They did not report any obvious side effects which was surprising as regulation of the actin cytoskeleton is a common pathway with essential roles throughout the body. This reminds of the situation in few hereditary diseases affecting the podocyte actin cytoskeleton. For instance, mutations in alpha-actinin-4, a gene expressed widely, results in a podocyte-specific phenotype as the only significant finding is albuminuria and progressive impairment of renal function (37). Thus, the actin cytoskeleton in podocytes seems to be more susceptible to dysregulation, which, on the other hand, can perhaps be reversed using actin-targeting drugs. Targeting actin-regulating molecules that are specific to podocytes, such as synaptopodin, can offer a more podocyte-targeted therapy option.

### Gprc5a

**Figure 2:**
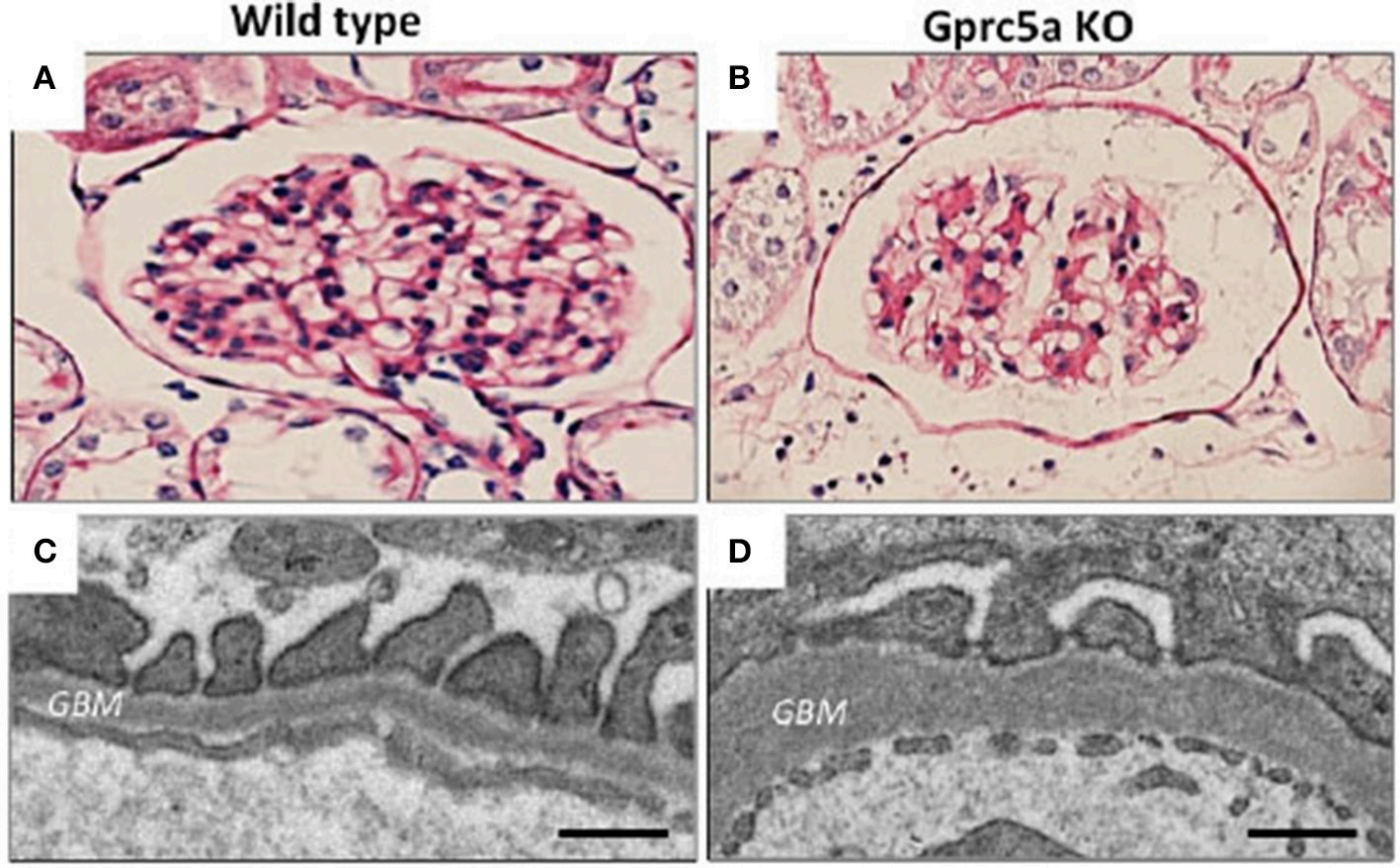
Glomerular pathology in Gprc5a-deficient mice. **(A,B)** Mesangial matrix is expanded in aging Gprc5a KO mice. **(C,D)** Electron microscopy reveals thickening of the GBM in Gprc5a-deficient mice, which is a hallmark sign of DN in humans. Magnifications: ×400 **(A,B)**. Scale bars: 300 nm **(C,D)**.

We recently identified an orphan G-protein coupled receptor (GPCR), Gprc5a, as a new highly podocyte-enriched protein that was significantly downregulated in patients with DN (38). Importantly, inactivation of Gprc5a in mouse phenocopied key histopathological features of human DN, including thickening of the GBM and mesangial expansion (Figure 2). Moreover, Gprc5a-deficient mice were more prone to develop nephropathy under diabetic conditions. Our studies suggest that Gprc5a-mediated signaling in podocytes has a pathogenic role in the progression of DN.

As a GPCR, Gprc5a, may represent a particularly interesting opportunity to directly target and pharmacologically manipulate podocyte signaling and health. However, Gprc5a is an orphan receptor that lacks the long N-terminal extracellular domain that is usually necessary for ligand binding and activation of downstream signaling via G-proteins of this class of receptor. In fact, we do not know today whether Gprc5a couples to G-proteins and to what intracellular signaling pathways it specifically couples and activates. Further studies are warranted to further understand the signaling role of this receptor in podocytes and whether manipulating such signaling may be podocyte protective.

The examples given above provide a representative sample of the data supporting the potential therapeutic efficacy that might be afforded by targeting the molecular machinery of the podocyte. In the section below, we describe some of the model systems used to enable the molecular and functional characterization of podocyte biology.

## Podocyte cell culture as a model system to discover new therapeutic compounds

**Figure 3:**
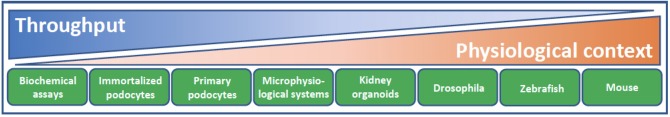
Model systems for podocyte drug discovery. The challenge of drug discovery and development lies in finding the balance between high throughput reductionist assay systems of limited physiological context and model systems with lower throughput but increasing physiological relevance and translation. Biochemical assays facilitate screening of a given drug target against a large library of compounds. Cellular context and target validation can be afforded using 2D podocyte cell culture and appropriate phenotypic readouts, such as actin cytoskeletal dynamics and apoptosis. Microphysiological systems that recapitulate the form and function of the glomerular filtration barrier, *ex vivo* glomeruli, kidney organoids and animal models from diverse species all lend increasing physiological complexity to target validation. As methodologies continue to evolve and improve in terms of their physiological context and throughput, there are increasing opportunities to accommodate these systems into a drug discovery cascade for identification of podocyte therapeutics.

Over the past few years, there has been a small burst in the number of publications describing the use of cultured podocytes in high content or phenotypic screening assays to facilitate the discovery of novel podocyte-specific hit compounds (39–43). Such cell-based assays trade the known inherent limitations of cell culture for the ability to screen large numbers of compounds for efficacy that would otherwise not be possible *in vivo* (Figure 3). With respect to the podocyte, recapitulation of the *in vivo* phenotypic characteristics of this cell type *in vitro* is particularly challenging. The *in vivo* podocyte is a highly differentiated cell type whose structure is exquisitely matched to its function; slender, interdigitating foot processes extend and enwrap the urinary side of the glomerular capillary wall and together with their bridging slit diaphragms form the most well-recognized cellular features of this cell (Figure 2). When cultured in isolation, it is well appreciated that podocytes typically undergo rapid de-differentiation, morphological simplification and loss of defined foot processes as well as slit diaphragms. Below, we provide some specific examples of significant developments in this field as well as in screening technologies.

Aside from establishing a robust screening assay that allows the filtering of compounds into positive and negative hits, selecting a disease-relevant phenotype with a corollary to human disease is also of crucial importance. To this end, high content imaging technology that can capture changes in the actin cytoskeleton and overall cellular morphology as well as more downstream readouts such as apoptosis have been put to use and are considered congruent readouts to what characterizes some of the changes seen in the podocyte in disease. In a collaborative effort, AstraZeneca and Evotec screened >120,000 compounds in a target-agnostic approach for their ability to afford protection to human podocytes from glucolipotoxicity-induced apoptosis (39). As expected, we identified a number of compounds with known targets (particularly kinases) to which an important role in regulating podocyte biology had previously been ascribed. More importantly however, we have also identified podocyte protective compounds to which the target-mediated mechanism is not known. Deconvoluting the target identity of such hits is a challenging undertaking but may ultimately lead to the discovery of novel podocyte therapeutics.

More recently, Sieber et al. designed a high-throughput screening assay to identify compounds that protect podocytes from thapsigargin-mediated ER stress and cell death (41). Rather than using such a large, incompletely annotated compound set as mentioned above, the authors screened a much smaller library of clinically applied and preclinicaly characterized molecules; among the hits identified were the BRAF inhibitor GDC-0879 and forskolin. Strikingly, additional studies demonstrated that both compounds were dose-response protective against a series of cellular stressors including adriamycin, staurosporine, palmitate, tunicamycin and brefeldin A. cAMP, the second messenger generated by forskolin-dependent activation of adenylate cyclase is part of a regulatory interconnected network signaling motif central to podocyte morphology and function and has been validated *in vivo* (44). The involvement of BRAF in podocyte survival *in vitro* is interesting and needs to be further understood considering that treatment with BRAF inhibitor, dabrafenib, associated with the development of nephrotic syndrome in melanoma patients and podocyte injury *in vitro* (45). BRAF inhibitors were not present in our selected compound screen but forskolin was and was not found to be protective against glucolipotoxicity-induced apoptosis. The possible reasons for these differences are likely many, including potential species differences (human vs. mouse podocytes), the use of immortalized cell lines, as well as differences in experimental design.

An alternative strategy to using immortalized podocyte cell lines takes advantage of a Nephrin-EGFP knockin mouse line and subsequent cultivation of glomeruli and primary podocytes (42, 43). Freshly isolated glomeruli that adhere to the plate and podocytes that migrate away from the glomerular core undergo dedifferentiation within days and are characterized by a loss of podocyte-specific marker gene expression, such as nephrin. Compounds that are able to maintain or promote GFP fluorescence can be screened automatically and considered as putative factors that oppose podocyte dedifferentiation. What this screening set up gives up in terms of throughput it gains with regards to using glomeruli/podocytes *ex vivo* that are as close as possible to the *in vivo* situation. Regardless of the screening platforms used, the validity of any podocyte-protective compounds identified in screening campaigns as bona fide physiological mediators specifically regulating podocyte health in disease models will require significant effort including proof that the putative target in podocytes is indeed the culprit.

Continued advances in cell culture techniques and the use of more complex assay systems to understand podocyte biology will continue to fuel the discovery pipeline. Although the immortalized mouse and human podocyte cell lines in use today have been fundamental tools to the podocyte research community, they are an imperfect surrogate with limited mimicry of the *in vivo* podocyte. Just how far one can push an isolated *in vitro* podocyte culture toward a molecular and structural phenotype that more completely recapitulates the *in vivo* podocyte with foot processes and slit diaphragms has been elegantly demonstrated using primary rat podocytes (46). Strikingly, when cells were grown in the presence of heparin and all-trans retinoic acid on laminin-coated plates, they were able to coax podocytes to project primary processes that further bifurcated and appeared to interdigitate with adjacent cells. Both the structural and podocyte-specific gene and corresponding protein expression patterns are remarkable when compared to uninduced podocytes. The authors note that they were unable to elicit a similarly high level of podocyte differentiation when they used mouse primary cell cultures and cell lines. Given the recent advances in derivation and generation of human pluripotent stem cells into podocyte-like cells (47), it should only be a matter of time before a much improved 2-D human podocyte cell culture system that combines techniques such as those described above will be developed and used for high content screening to find new podocentric therapies.

Overcoming the limitations of a reductionist approach utilizing isolated podocytes in culture for target identification and validation can also be envisaged by looking at the significant strides made in generating kidney organoids (48, 49), microphysiological systems (50) and recellularized kidneys (51). Pluripotent stem cells can be stimulated to differentiate along the mesoderm lineage and subsequently directed to give rise to multiple nephron cells types which self-organize into spheroid structures comprised of spatially-segregated and distinct glomerular and tubular segments. Podocyte-like cells expressing nephrin are especially well represented and organoids clearly model many aspects of renal development and structure. Interestingly enough, these organoids have shown promise to act as a model system to study kidney disease. Whereas generation of kidney organoids from stem cells genetically engineered using CRISPR/Cas9 genome-editing to knockout the polycystic kidney disease genes gave rise to cyst formation in tubule structures, knockout of podocalyxin (a highly expressed podocyte gene) caused defects to the podocyte-like cells as characterized by disrupted junctional complexes. This area of research is moving fast and we will undoubtedly see considerable advances in the years to come as other gene targets are evaluated.

Podocytes and endothelial cells lay down the basement membrane that is sandwiched between these two cell types and all together, form the trilaminar GFB. Recapitulating such an arrangement *in vitro* is the ultimate goal of bioengineers and requires not only the two highly differentiated cellular components but also a matrix support and separated flow chambers that allow pressure and shear stress forces to be manipulated in a manner that mimics the biophysical properties of the GFB *in vivo*. The most recent example of such a device is an excellent illustration of such an integrative approach and highlights just how far the field has come (50). Although throughput for compound screening using such a model system is limited, it holds a great deal of promise as it can be utilized to study a structurally and functionally intact GFB.

## Animal models for podocyte screening

Moving beyond *in vitro* models and into more complex animal models such as the fruit fly and zebrafish also has significant merit as a more holistic approach to facilitate the discovery and testing of new podocyte therapeutics Despite coming from vastly distinct phyla and lacking a defined kidney *per se*, the Drosophila nephrocyte is astoundingly similar at both a structural and molecular level to the mammalian podocyte and has been used to model monogenic forms of nephrotic syndrome (52). The speed and amenability to genetic manipulation make the fruit fly an attractive option for unbiased evaluation of the impact of loss-of-function gene studies. Speaking further to the basis for evolutionary conservation, the Drosophila nephrocyte also is a model to study DN; chronic high dietary sucrose-fed flies display nephrocyte dysfunction and a decrease in the expression of the mammalian ortholog of nephrin and phenocopy many aspects of mammalian DN (53).

The zebrafish pronephros is also an exceptional model system for studying podocyte biology and glomerular function during the developmental phase owing to the transparency of the zebrafish embryo and ability to visually assess glomerular dysfunction by presence of overt cardiac edema. Morpholino knockdown of gene expression has facilitated rapid screening of putative podocyte gene function in maintaining an intact and functional GFB and often phenocopies the situation observed in rodent models. More recently, the generation of stable mutant lines using TALENs and CRISPR/Cas9 has been utilized to complement morpholino-generated data and avoid morpholino technology-biased artifacts (54). The generation of transgenic zebrafish models that incorporate inducible podocyte injury with a fluorescent tracer for proteinuria raise the possibility to potentially screen for compounds that can improve podocyte health *in vivo* (55). Nephrotoxic compounds, including puromycin and adriamycin, routinely used toxins to induce nephrotic syndrome and FSGS in rodents, also appear useful in eliciting podocyte-specific injury when used at suitable doses that minimally affect other organ systems and embryo development (56). Moreover, development of transgenic lines in which different glomerular cell types can be visualized with fluorescent reporters provides a read-out that can be useful in high throughput studies.

## The future of podocyte-tailored therapies

As mentioned earlier, ACE inhibitors and ARBs, first developed for their beneficial systemic cardiovascular effects, form the foundation of today's standard of care treatment for patients with albuminuria and DN. However, the beneficial effects of this intervention on kidney disease progression have only later been understood to arise, to a large part, as a result of a renal-intrinsic mechanism of action originating from the ability of this class of drugs to directly modulate and improve glomerular hemodynamics. Some 30 years have passed since the utility of this drug class in treating patients with CKD was established and since that time no new drugs for treating CKD have been approved. Repurposing of drugs initially designed for other indications has formed much of the strategic approach to identifying novel CKD therapeutics but this strategy has not proved successful. The renal community was hopeful that Atrasentan, an endothelin A receptor-selective antagonist, would prove effective in treating DN patients, but disappointingly, the SONAR trial was stopped early due to a lack of events related to the primary outcome (Abbvie media statement). Also very recently, the UK HARP-III trial designed to assess renal efficacy of Entresto (a combined ARB and neprilysin inhibitor), was shown to be without significant improvement on ACR and GFR vs. Irbesartan (ASN 2017 poster FR-PO1064). Perhaps the most interesting and promising example of drug repurposing comes from the SGLT2 class of inhibitors designed to facilitate blood glucose lowering in diabetes by preventing glucose reabsorption along the proximal tubule and promoting glucosuria. In addition to its glucose lowering activity, its kidney-specific MoA speaks in favor of a direct renal intrinsic mechanism where tubuloglomerular feedback is activated secondary to reduced distal tubule delivery of sodium. In this manner, a number of clinical trials are underway to determine whether SGLT2 inhibitors might become the first kidney-targeted therapeutic that is beneficial in treating CKD.

As described earlier in this review, ample preclinical evidence supports the therapeutic potential of targeting the podocyte. Moving from proof of concept in rodent models of disease to clinical evaluation still has a long way to go. The molecular mechanisms of podocyte dysfunction in disease continue to be unraveled and there is reason to be optimistic that with continued research, novel therapeutic targets will be identified. FSGS orphan indications will hopefully pave the way to validating the clinical benefit of targeting unique molecular entities expressed by the podocyte. The hope and vision is that this will then pave the way to an expanded study of targeting the podocyte in DN.

## Author contributions

All authors listed have made a substantial, direct and intellectual contribution to the work, and approved it for publication.

### Conflict of interest statement

ML is employed by AstraZeneca. JP's research is supported by AstraZeneca.
